# Proteomic and phosphoproteomic analysis of renal cortex in a salt-load rat model of advanced kidney damage

**DOI:** 10.1038/srep35906

**Published:** 2016-10-24

**Authors:** Shaoling Jiang, Hanchang He, Lishan Tan, Liangliang Wang, Zhengxiu Su, Yufeng Liu, Hongguo Zhu, Menghuan Zhang, Fan Fan Hou, Aiqing Li

**Affiliations:** 1 State Key Laboratory of Organ Failure Research, National Clinical Research Center of Kidney Disease, Division of Nephrology, Nanfang Hospital, Southern Medical University, Guangzhou, China; 2 The First People’s Foshan Hospital, Foshan, China; 3 Division of Nephrology, First Affiliated Hospital of Guangzhou University of Traditional Chinese Medicine, Guangzhou 510405, P.R. China

## Abstract

Salt plays an essential role in the progression of chronic kidney disease and hypertension. However, the mechanisms underlying pathogenesis of salt-induced kidney damage remain largely unknown. Here, Sprague-Dawley rats, that underwent 5/6 nephrectomy (5/6Nx, a model of advanced kidney damage) or sham operation, were treated for 2 weeks with a normal or high-salt diet. We employed aTiO_2_ enrichment, iTRAQ labeling and liquid-chromatography tandem mass spectrometry strategy for proteomic and phosphoproteomic profiling of the renal cortex. We found 318 proteins differentially expressed in 5/6Nx group relative to sham group, and 310 proteins significantly changed in response to salt load in 5/6Nx animals. Totally, 1810 unique phosphopeptides corresponding to 550 phosphoproteins were identified. We identified 113 upregulated and 84 downregulated phosphopeptides in 5/6Nx animals relative to sham animals. Salt load induced 78 upregulated and 91 downregulated phosphopeptides in 5/6Nx rats. The differentially expressed phospholproteins are important transporters, structural molecules, and receptors. Protein-protein interaction analysis revealed that the differentially phosphorylated proteins in 5/6Nx group, Polr2a, Srrm1, Gsta2 and Pxn were the most linked. Salt-induced differential phosphoproteins, Myh6, Lmna and Des were the most linked. Altered phosphorylation levels of lamin A and phospholamban were validated. This study will provide new insight into pathogenetic mechanisms of chronic kidney disease and salt sensitivity.

The prevention and treatment of chronic kidney disease has become one of the important public health problems in the world^1^, and advanced kidney damage with a severe decrease in the glomerular filtration rate is likely to be accompanied with the complications of kidney disease such as high blood pressure, anemia, bone disease, and cardiovascular diseases. Most advanced kidney damage patients suffer from varying degrees of hypertension, and persistent high blood pressure will accelerate the progress of chronic kidney disease, creating a vicious cycle. Therefore, actively controlling blood pressure has important implications for the treatment of advanced kidney damage^2^.

Salt is one of the most important environmental factors that promote the development of hypertension^3^. Since 1976, it has been well recognized that patients and animal models with advanced kidney damage are often salt sensitive^4^,^5^. Further, salt plays an essential role in the progression of chronic kidney disease, and prolonged salt load was known to cause renal injury including proteinuria, renal fibrosis and renal hypertrophy, in blood pressure–dependent and –independent ways^6^,^7^. Dietary salt restriction has been shown to reduce albuminuria, renal fibrosis and blood pressure^8^. However, the mechanisms underlying the pathogenesis of salt-induced kidney damage remain largely unknown.

Protein phosphorylation plays a key role in many cellular events including metabolism, secretion, homeostasis, transcriptional and translational regulation, and cellular signaling^9^. There is some evidence that phosphorylation of proteins and related phosphorylation events are essential to regulate renal signaling pathways such as transforming growth factor (TGF)-10, lysine deficient protein kinase (WNK)-11, arginine vasopressin (AVP)-associated ones^12^, which participate in the maintenance of renal function and the development of kidney diseases. However, the molecular details in such a process are not well understood. Hence, identifying phosphoproteins and phosphorylated molecules that might contribute to the salt sensitivity using large-scale and high throughput approaches may provide us an insightful understanding of salt sensitivity.

Mass spectrometry (MS)-based proteomics in combination with phosphoprotein enrichment technique has enabled us to globally analyze intracellular signaling events as well as the common post-translational modification-phosphorylation without prior knowledge of function or distribution^13^. Isobaric tags for relative and absolute quantification (iTRAQ) is a stable isotope-labeled, relative and absolute quantitative proteomics technology, with high accuracy and precision in quantification, higher repeatability and simultaneously quantitative analysis on multiple samples. Furthermore, due to the new stable isobaric labeling technique, iTRAQ has been applied more widely to study post-translational modifications than other proteomic approaches^14^. Several studies have been performed on quantitative phosphoproteomics of renal proximal and distal tubule^15^, renal collecting duct cells^16^, or renal thick ascending limb cells^17^. However, there are so far no phosphoproteomics studies on salt-induced kidney injury. Therefore, in this study, we aim to investigate proteomic and phosphoproteomic changes of renal cortex in a rat model of advanced kidney damage in response to salt load by using iTRAQ technology and TiO_2_ phosphopeptide enrichment method^18^ coupled with tandem MS [liquid chromatography (LC)-MS/MS]^19^,^20^,^21^.

## Result

### Changes in physiological parameters during salt load in CRF rats

To confirm successful preparation of 5/6Nx rat models, we examined body weight, systolic blood pressure, serum creatinine, and 24 h urinary protein excretion of 5/6 nephrectomy or sham operated rats. As shown in Supplementary Table S1, sham and 5/6Nx rats showed similar development of body weight after 10 weeks of 5/6 nephrectomy or sham operation. The 5/6Nx rats showed significantly higher systolic blood pressure, serum creatinine and 24 h urine protein excretion when compared with sham group. After 2 weeks of high salt diet treatment, kidney weight/body weight and systolic blood pressure increased significantly with increasing dietary salt content in 5/6Nx group. In addition, high salt load resulted in significant increase of 24 h urinary sodium excretion and urinary protein excretion in 5/6Nx rats (Table 1).

### Analysis of proteins associated with salt load and renal failure

In this study, we carried out proteomic profiling of renal cortex from sham and 5/6Nx rats fed normal or high salt diet, using a highly sensitive iTRAQ-coupled LC-MS/MS method. A total of 2695 peptides were unambiguously identified. The complete data report for all identified proteins can be viewed in Supplementary Dataset 1. The distribution of the log_2_(5/6Nx + NS)/(Sham + NS) or (5/6Nx + HS)/(5/6Nx + NS) ratio for abundance of all identified proteins was plotted as a histogram (Supplementary Figures S1A and 2B). Most of the log2 ratios for the proteins cluster in a normal distribution centered around zero, which corresponded to a (5/6Nx + NS)/(Sham + NS) or (5/6Nx + HS)/(5/6Nx + NS) ratio of ~1.

Among these identified proteins (Supplementary dataset 1), we found 318 proteins differentially expressed in (5/6Nx + NS)/(Sham + NS) comparison group, including 164 upregulated and 154 downregulated. Comparing proteins from 5/6Nx + HS group versus those from 5/6Nx + NS group, there were 310 proteins significantly changed including 157 increased and 153 decreased in abundance. Relative quantification of significantly altered proteins in (5/6Nx + NS)/(Sham + NS) and (5/6Nx + HS)/(5/6Nx + NS) comparison groups were shown as a log_1.5_(5/6Nx + NS)/(Sham + NS) or (5/6Nx + HS)/(5/6Nx + NS) ratio (Supplementary Figure S1C,D). Due to space limitation, an expanded list of significantly changed proteins with a cut-off value of 1.5-foldup- or down-regulation was shown in Table 2.

### Identification of phosphopeptides, phosphosites and phosphoproteins

**Figure 1:**
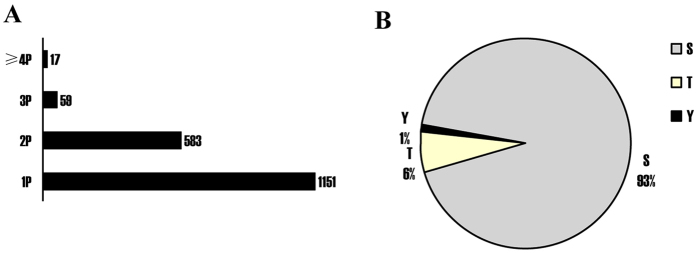
Characterization of phosphopeptides and phosphosites at the Log_2_ (5/6Nx + NS)/(Sham + NS) or Log_2_(5/6Nx + HS)/(5/6Nx + NS) ratio in (5/6Nx + NS)/(Sham + NS) and (5/6Nx + HS)/(5/6Nx + NS) comparison groups. (**A**) Distribution of singly, doubly, triply and quadruply phosphorylated peptides; (**B**) Distribution of the Ser, Thr, Tyr phosphosites in singly phosphorylated peptides. Phosphopeptides were categorized into class I phosphosites by calculating the probabilities for phosphorylation at each site based on posttranslational modification scores. Here, only class I phosphosites (high probability) were used to analyze the distribution. 1P, 2P, 3P and 4P represent singly, doubly, triply and quadruply phosphorylated peptides, respectively. Sham + NS, sham operation + normal salt; 5/6Nx + NS, 5/6Nx + normal salt; 5/6Nx + HS, 5/6Nx + high salt.

We applied MS-based iTRAQ quantitative proteomics in combination with phosphopeptide enrichment method to identify phosphopeptides and phosphoprotein and quantify their expression level. Totally, 1911 phosphorylated peptides were identified; among them 1810 phosphopeptides have quantitative information, which corresponded to 550 phosphoproteins. The distribution of (5/6Nx + NS)/(Sham + NS) or (5/6Nx + HS)/(5/6Nx + NS) ratio for phosphopeptide abundance was plotted as a histogram (Supplementary Figure S2A,B). More than 80% of the total number of phosphopeptide identified cluster in a normal distribution centered at the log2 ratio of ~0, which corresponded to a (5/6Nx + NS)/(Sham + NS) or (5/6Nx + HS)/(5/6Nx + NS) ratio of ~1. To precisely assign phosphorylation sites from tandem mass spectra, posttranslational modification score was used to estimate the probability of correct phosphorylation site as described previously^22^. Totally, 2565 phosphosites were localized with high confidence. The percentages of singly, doubly, triply, and more highly phosphorylated peptides were 63.6%, 32.23%, 3.26% and 0.94%, respectively (Fig. 1A).The phosphosites of singly-phosphorylated peptides included 1% phosphotyrosine site, 6% phosphothreonine site, and 93% phosphoserine site (Fig. 1B).

### Differentially phosphorylated peptides in response to CRF and salt load

The identified phosphopeptides were shown in detail in Supplementary Dataset 2. It was found that 197 peptides corresponding to 149 phosphorylated proteins were differentially phosphorylated, i.e., 113 increased and 84 decreased phosphorylation level in 5/6Nx rats relative to sham ones fed normal salt diet ((5/6Nx + NS)/(Sham + NS)) (Supplementary Figure S2C). In the comparison group of high salt-fed CRF rats versus normal salt-fed 5/6Nx animals, 169 peptides (78 increased, 91 decreased) were differentially phosphorylated corresponding to 127 phosphoproteins (Supplementary Figure S2D). To further display the differentially phosphorylated proteins coordinatially regulated by advanced kidney damage and high salt intake, we made a list of these phosphorylated proteins in Supplementary Dataset3 and Venn diagram analysis of these proteins (Supplementary Figure S2E). Hierarchically clustered heatmaps of the differentially phosphorylated peptides for (5/6Nx + NS)/(Sham + NS) (Supplementary Figure S3) and (5/6Nx + HS)/(5/6Nx + NS) (Supplementary Figure S4) comparison groups showed that the differentially phosphorylated peptides were coordinately regulated by 5/6Nx and high salt intake. These complete lists of phosphopeptides, phosphosites and their abundance differences were presented in Supplementary Dataset 2. In addition, since proteinuria is an important marker of progression of renal disease and a strong predictor of morbidity, we have mined the complete list of phosphopeptides for podocyte-specific phosphoproteins, which are significantly more highly expressed in podocytes than nonpodocyte glomerular cells, based on previous studies^23^,^24^. The 23 podocyte-specific phosphoproteins were presented in Supplementary Dataset 4, which included tight junction protein ZO-1, Synaptopodin, Desmin, Vimentin, Voltage-dependent anion-selective channel protein 1, Clathrin heavy chain 1, PDZ and LIM domain protein 4, etc.

**Figure 2:**
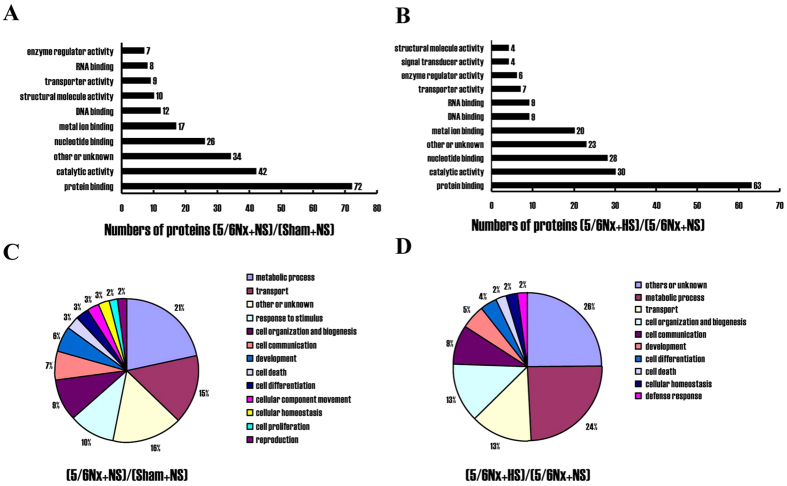
Quantification and Gene Ontology analysis of differentially phosphorylated peptides and proteins in (5/6Nx + NS)/(Sham + NS) and (5/6Nx + HS)/(5/6Nx + NS) comparison groups. Gene Ontology analysis of differentially expressed phosphoproteins based on their molecular function in (5/6Nx + NS)/(Sham + NS) (**A**) and (5/6Nx + HS)/(5/6Nx + NS) (**B**) comparison groups using PANTHER classification. (C & D) Gene Ontology analysis of differentially expressed phosphoproteins based on biological process with PANTHER program. Sham + NS, sham operation + normal salt; 5/6Nx + NS, 5/6Nx + normal salt; 5/6Nx + HS, 5/6Nx + high salt.

To understand the roles of these differential phosphorylated proteins in development of 5/6Nx and their association with high salt intake, a Gene Ontology analysis with PANTHER (Protein ANalysis THrough Evolutionary Relationships) system was used to categorize the biological information including molecular functions and biological process. Gene Ontology analysis for (5/6Nx + NS)/(Sham + NS) comparison group (Fig. 2) showed that the 149 differentially phosphorylated proteins were categorized into 10 functional groups including protein binding, catalytic activity, nucleotide binding, etc. Based on their biological process (Fig. 2A,C), these proteins were categorized into 13 groups such as metabolic process, transport, response to stimulus, etc. Similarly, 127 differentially phosphorylated proteinsfor (5/6Nx + HS)/(5/6Nx + NS) comparison group were categorized into11 functional groups including protein binding, catalytic activity, nucleotide binding, metal ion binding, etc (Fig. 2B). Based on their biological process, these phosphoproteins are classified into 10 groups such as metabolic process, transport, cell communication, and cell organization and biogenesis (Fig. 2D).

### Signaling pathways involved in CRF and salt load

**Figure 3:**
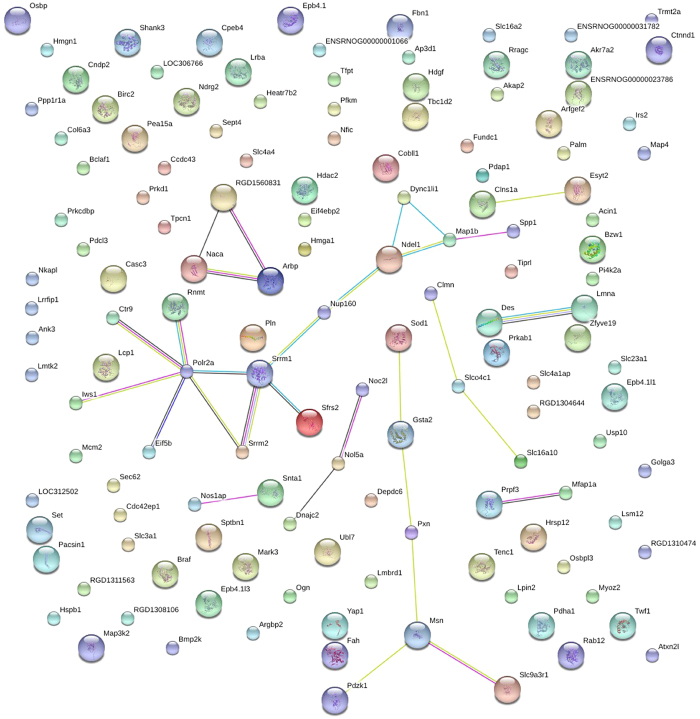
STRING analysis reveals protein interaction networks in renal phosphoproteome in (5/6Nx + NS)/(Sham + NS) comparison group. Interactions of the identified phosphoproteins were mapped by searching the STRING (Search Tool for the Retrieval of Interacting Genes/Proteins) database version 9.0 with a confidence cutoff of 0.6. In the resulting protein association network, proteins are presented as nodes which are connected by lines whose thickness represents the confidence level (0.6–0.9). Sham + NS, sham operation + normal salt; 5/6Nx + NS, 5/6Nx + normal salt.

**Figure 4:**
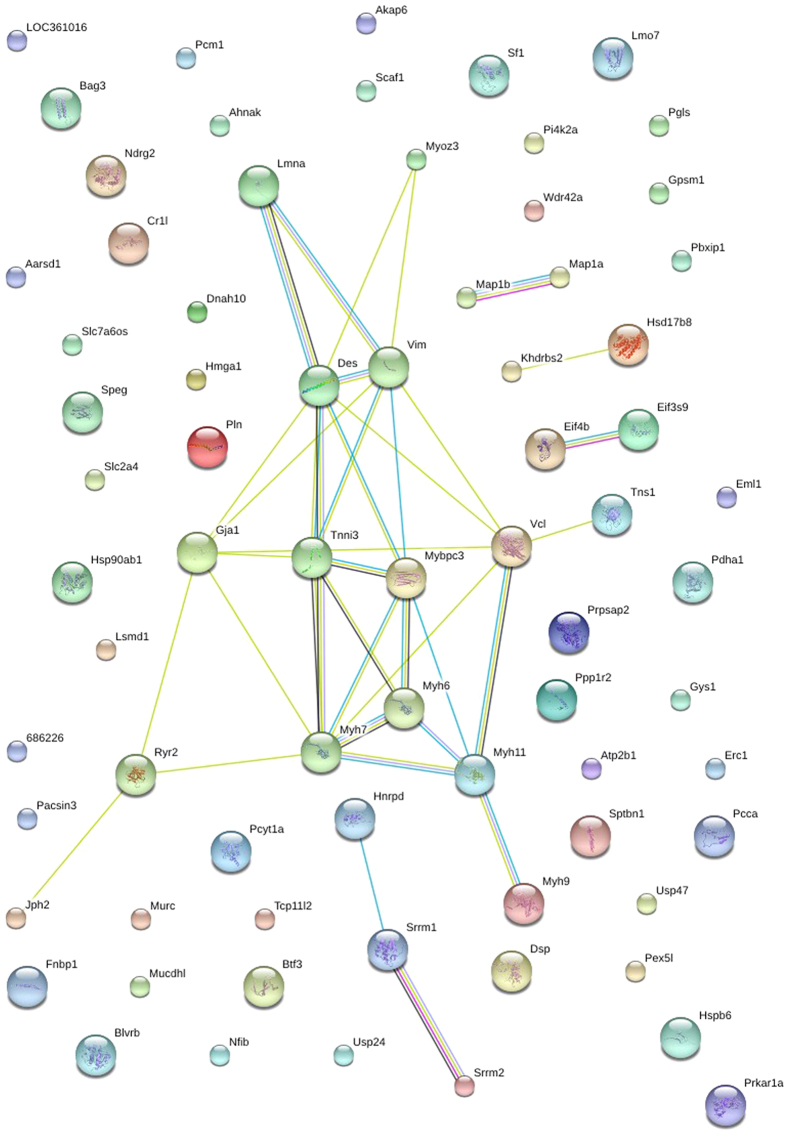
STRING analysis reveals protein interaction networks in renal phosphoproteome in (5/6Nx + HS)/(5/6Nx + NS) comparison group. Interactions of the identified phosphoproteins were mapped by searching the STRING database version 9.0 with a confidence cutoff of 0.6. In the resulting protein association network, proteins are presented as nodes which are connected by lines whose thickness represents the confidence level (0.6–0.9). 5/6Nx + NS, 5/6Nx + normal salt; 5/6Nx + HS, 5/6Nx + high salt.

STRING (Search Tool for the Retrieval of Interacting Genes) is a meta-database program that generates a network of protein interactions from high-throughput experimental data, literature, and predictions based on genomic context analysis^25^. The networks formed by interacting proteins are helpful in understanding potential molecular mechanisms of advanced kidney damage and the effects of salt on renal failure. Here, the STRING analysis showed that 36 significantly regulated phosphoproteins in (5/6Nx + NS)/(Sham + NS) comparison group were functionally linked (Fig. 3), and the most linked phosphoproteins were Polr2a, Srrm1, Srrm2, Msn, Ndel1, Map1b, Pxn, Gsta2, Nol5a, Naca, Arbp, RGD1560831, etc. In HC/NCcomparison group, 24 significantly regulated phosphoproteins were functionally linked, and the most connected protein nodes were Myh6, Myh7, Myh11, Lmna, Des, Vim, Tnni3, Mydpc3, Gja1, Vcl, Ryr2 and Myoz3 (Fig. 4).

To assign potential signaling pathways involved in the renal phosphoproteome, the identified phosphoproteins were searched against well-known pathway database, Kyoto Encyclopedia of Genes and Genomes (KEGG)^26^,^27^. Several KEGG pathways were obtained for differentially phosphorylated proteins in (5/6Nx + NS)/(Sham + NS) comparison groups, such as insulin signaling pathway, adipocytokine signaling pathway, hypertrophic cardiomyopathy and focal adhesion (Supplementary Dataset 5).

The biological pathways obtained from differentially phosphorylated proteins in HC/NC comparison groups include damyotrophic lateral sclerosis, mTOR signaling pathway, purine metabolism, spliceosome and insulin signaling pathway (Supplementary Dataset 6). Endocrine and stress-related pathways are commonly activated under conditions of 5/6Nx. Proteins regulated by these pathways included acetyl-Coenzyme A carboxylase beta, acidic ribosomal phosphoprotein P0, adaptor-related protein complex 3, delta 1 subunit, desmin, erythrocyte protein band 4.1-like 1, erythrocyte protein band 4.1-like 3, fumarylacetoacetate hydrolase, glutathione S-transferase A2, insulin receptor substrate 2, mitogen activated protein kinase kinase kinase 2, paxillin, phosphofructokinase, phospholamban, polymerase (RNA) II polypeptide A, procollagen, type VI, AMP-activated protein kinase, protein phosphatase 1, mTORC, ribosomal protein S3, similar to Histone deacetylase 2 and superoxide dismutase 1.

### Validation of selected proteins and analysis of their downstream gene expression

**Figure 5:**
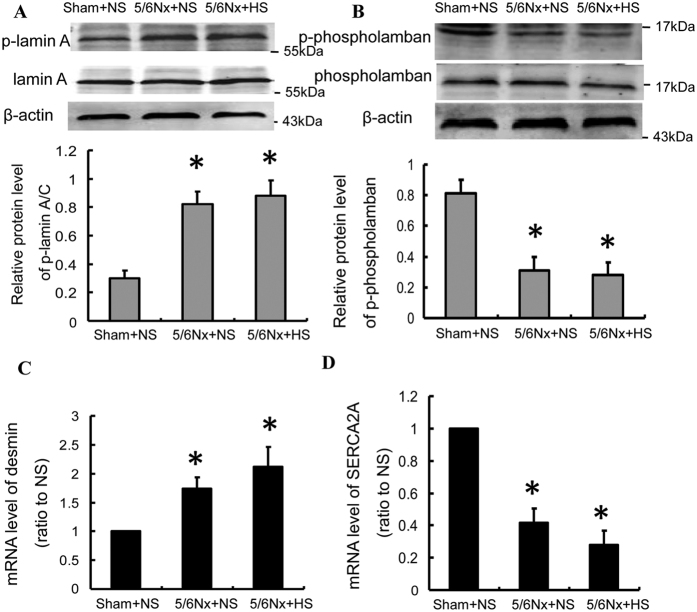
Phosphoryation levels of lamin A and phospholamban as well as expression of their downstream genes desmin and SERCA2a significantly increased in 5/6Nx rats. (**A,C**) Protein level of p-lamin A (**A**) and mRNA level of its downstream gene desmin (**C**) significantly increased in 5/6Nx + NS and 5/6Nx + HS groups. (**B,D**) Phosphorylation level of phospholamban decreased (**B**) and that resulted in decrease of mRNA level of the downstream gene SERCA2a (**D**) in 5/6Nx + NS and 5/6Nx + HS groups. Data are expressed as the mean ± SD of three independent experiments (n = 6 in each group). *p < 0.05 *vs.* Sham + NS, sham operation + normal salt; 5/6Nx + NS, 5/6Nx + normal salt; 5/6Nx + HS, 5/6Nx + high salt.

We analyzed two differentially phosphorylated proteins phospho-lamin A and phospho-phospholamban induced by high salt diet in 5/6Nx group, as well as their downstream genes desmin and SERCA2a^28^,^29^. Initially, western blot analysis of phospho-lamin A expression was performed. Consistent with our proteome analysis, a significant increase in phospho-lamin A expression was observed in 5/6Nx + NS and 5/6Nx + HS groups as compared with NS group (Fig. 5A). Further, a significant increase in desmin mRNA level was observed in 5/6Nx + NS and 5/6Nx + HS groups (Fig. 5C), that matched increased phospho-lamin A expression. Expression of phospho-phospholamban in renal cortexes was then analyzed. Western analysis showed a marked decrease in phosphor-phospholamban expression in 5/6Nx animals fed normal or high salt diet (Fig. 5B). Consistently, realtime PCR analysis revealed a decrease in mRNA levels of its downstream gene SERCA in 5/6Nx + NS and 5/6Nx + HS groups (Fig. 5D).

## Discussion

Studies in humans have shown that salt intake increases the amount of urinary protein, which is a major risk factor for developing kidney disease and cardiovascular disease^30^. In our previous study, we had profiled a large cardiac phosphoproteome data in salt-load 5/6Nx rat model^18^. Although a growing body of evidence demonstrated that high dietary salt loading exerts detrimental cardiac effects, the kidneys are the most severely affected organs by salt intake in patients and animals with chronic kidney disease. Proteomic technologies are used with increasing frequency in renal research. Phosphoproteomic analysis has been conducted on renal cortex^15^, different segments of tubules^15^ and renal cells^17^,^16^, whereas no studies have used these techniques to profile pathway-specific signaling responses to salt load in CRF patients or animals. In this study, we applied the MS/MS spectra by combining phosphopeptide enrichment with MS-based iTRAQ quantitative proteomics to profile phosphoproteome of renal cortex in 5/6Nx rats in response to high salt intake. Although the cardiac responses to salt loading had been investigated by a proteomic approach^18^, this study focused on kidneys, the major organ responsible for maintenance of body water and salt balance. Here, we found 318 proteins differentially expressed in 5/6Nx relative to sham group, and 310 proteins significantly changed in response to salt load in 5/6Nx animals. A total of 1810 unique phosphorylation peptides were identified and quantified, corresponding to 550 phosphorylated proteins. It is worth pointing out that this study presents many previously unreported phosphorylated sites on a number of transporters critical to renal transport physiology, which significantly extended the known cortical membrane proteome.

There were 197 peptides differentially phosphorylated, during normal dietary salt feeding in 5/6Nx animals compared with sham ones, including 113 increased and 84 decreased in phosphorylation level; 169phosphopeptides were differentially expressed in high-salt fed 5/6Nx rats relative to normal-salt fed ones, including 78 increased and 91 decreased. By using Panther online database, we conducted Gene Ontology classification for the phosphorylated proteins which corresponded to differentially phosphorylated peptides. The phosphorylated proteins in both (5/6Nx + NS)/(Sham + NS) and (5/6Nx + HS)/(5/6Nx + NS) groups are involved in metabolism, cell composition, proliferation, differentiation, cell death and other biological processes, wherein metabolism occupies the biggest portion, followed by the transport process. These indicate that the occurrence and development of advanced kidney damage and salt sensitivity is one of complex extensive adjustment modes.

Protein-protein interactions play a central role in many cellular functions, such as chemical transport, signal transduction and metabolism, therefore interaction analysis on these key molecules will help us to better understand precise pathomechanism of salt-induced advanced kidney damage. By using STRING database, differentially phosphorylated proteins in both (5/6Nx + NS)/(Sham + NS) and (5/6Nx + HS)/(5/6Nx + NS) groups were further analyzed. The STRING database not only reveal direct, physical interactions of two proteins, but also links proteins because they exhibit a genetic interaction or are known to catalyze subsequent steps in a metabolic pathway^25^. Some proteins identified in this study have been known to be regulated by 5/6Nx. Analysis of differentially phosphorylated proteins in (5/6Nx + NS)/(Sham + NS) group showed that Srrm1 and Srrm2 were among the most functionally linked proteins, which were phosphorylated, glomerulus expressed proteins^24^. Phosphorylated Srrm1 has been reported to increase in abundance in response to a vasopressin analog in renal collecting duct cells^31^. Previous study reported that Pxn interacts with proteins that have a different localization as date hubs, and is involved in TGF beta1-driven epithelial-mesenchymal transition of renal epithelial cells^32^. As an antioxidant, NACA could prevent cell death and contrast-induced nephropathy by blocking the p38 MAPK/iNOS signaling pathway^33^. In a rat model of unilateral ureteral obstruction, progressive reduction of Gsta2 via Nrf2-Keap1 cellular defense pathway was demonstrated after 10 days of obstruction, suggesting impaired ability to mount the biological response to the prevailing oxidative stress leading to renal injury^34^. Altogether, these data suggested that these proteins are probably important targets of signaling pathways for the development and progression of kidney damage.

Among differentially phosphorylated proteins in (5/6Nx + HS)/(5/6Nx + NS) group, 24 proteins were functionally linked, that are likely signaling molecules involved in development of salt sensitivity in advanced kidney damage. The most linked proteins included Myh6, Myh7, Myh11, Lmna, Des, Tnni3, Mydpc3 and Vcl, revealing that these molecules may be important targets of signaling pathways involved in salt sensitivity in advanced kidney damage. Myosin and its phosphorylation play an important role in cytokinesis^35^. Myosin phosphorylation is important for the normal recruitment of myosin molecules into the contractile ring structure. Changes of Myh 6, Myh7 and Myh11 phosphorylation suggest that dysregulation of cytokinesis may be associated with salt sensitivity in chronic kidney disease.

Between (5/6Nx + NS)/(Sham + NS) comparison and (5/6Nx + NS)/(5/6Nx + NS) comparison, we found that some proteins showed different trend of regulation, i.e., up-regulated in (5/6Nx + NS)/(Sham + NS) comparison, but down-regulated in (5/6Nx + HS)/(5/6Nx + NS) comparison and vice versa. This may be attributed to the different mechanisms underlying salt-induced renal injury and subtotal nephrectomy-induced renal failure. On one hand, high salt intake exerts direct effects to cause renal damage, such as affecting endothelial funcion, stimulating cardiotonic steroids, exerting profibrotic effects, and inducing paradoxical activation of the aldosterone receptor; on the other hand, it affects blood pressure, proteinuria and glomerular hemodynamics, consequently leading to renal damage^36^,^37^. However, reduction of renal parenchyma after subtotal nephrectomy results in adaptive single nephron hyperfiltration, which consequently leads to increase in glomerular capillary plasmaflow and hydraulic pressure. These finally contributed to intact nephron hypertrophy and sclerosis. Altogether, although salt treatment supposes to promote renal injury, the different pathogenic mechanisms of salt treatment and subtotal nephrectomy may explain the different trend of some proteins in (5/6Nx + NS)/(Sham + NS) comparison and (5/6Nx + HS)/(5/6Nx + NS) comparison.

Among the KEGG pathways we analyzed, insulin signaling pathway and adipocytokine signaling pathway were known to be associated with progression of chronic kidney disease^38^,^39^. Upregulation of proinflammatory cytokines (TNF-α, TGF-β, IL-6 etc) and reactive oxygen species substances, terminally deteriorated kidney injury in several experimental models^40^,^41^,^42^,^43^,^44^. Moreover, inhibition of mTOR with sirolimus apparently ameliorated glomerular hypertrophy, decreased the inflammation and fibrosis in tubular interstitial^45^,^46^,^47^,^48^,^49^. All these evidences revealed that insulin resistance, adipocytokines and mTOR signal pathway were involved the pathological mechanism of chronic kidney disease. Furthermore, acceleration of progress of renal damage with a high salt diet has been shown in several experimental models of renal disease and human^30^,^37^. Erk1/2 and p38 MAPK were activated by increased dietary salt intake, which further elevated oxidative stress and generation of TGF-β1 in kidney^50^,^51^,^52^. It indicated that MAPK signal pathway activation promoted the high salt dietary induced the deterioration of chronic kidney disease.

In order to identify phosphoproteins that may play a role in salt-induced kidney damage, we examined changes in phosphorylation levels of lamin A and phospholamban as well as their downstream genes desmin and SERCA2a, which altered significantly in response to high salt intake in 5/6Nx rats. It has been shown that activation of insulin signaling pathway can lead to phosphorylation of lamin A^53^. Alteration of insulin signaling pathway plays an important role in the pathogenesis of podocyte malfuncion and microalbuminuria^43^. These indicate that the phosphorylation of lamin A maybe involved in the pathogenic mechanism of advanced kidney damage. Phospholamban has a central role in modulating Ca^2+^ homeostasis and mediates development of cardiomyopathy^54^. In addition, elevated Nox2 activity increases phospholamban phosphorylation and 5/6Nx rats showed apparent oxidative stress with elevated renal Nox2 activity^55^,^56^. Thus, phospho-phospholamban may play a role in progression of chronic kidney disease.

Proteinuria is known as a key prognostic factor for renal failure. Podocytes constitute the final filtration barrier in the glomerulus, and their dysfunction plays a critical role in proteinuria. Thus, we generated a list of 23 phosphoproteins which was known to be specially expressed in podocytes based on studies by Rinschen *et al.*^23^ and Boerries *et al.*^24^. Several proteins have been previously linked to renal disease in man or model organisms, including slit diaphragm-associated protein ZO-1, vimentin and desmin, a marker of podocyte damage^57^,^58^. It has been known that endocrine or stress pathways such as insulin resistance or oxidative stress pathways are activated during chronic renal failure^59^. Insulin receptor substrate 2 (IRS-2) has been implicated as essential signaling intermediates in insulin-regulated glucose homeostasis. Elevated free fatty acids and oxidative stress, which plays a key role in development and progress of chronic kidney diseases, have been shown to promote the negative serine phosphorylation of the IRS protein and interfere with IR/IGF-1R signaling^60^,^61^. The family erythrocyte protein band 4.1 proteins are abundantly expressed in adrenal gland and have multiple functions in endocrine functions of adrenal gland^62^. A study using spontaneously hypertensive rats showed that fumarylacetoacetate hydrolase played an important role in glucose metabolism and defense against oxidative stress^63^. Disruption of acidic ribosomal phosphoprotein P0 leads to reactive oxygen species accumulation^64^.

In conclusion, we found 318 proteins (164 upregulated, 154 downregulated) differentially expressed in 5/6Nx relative to sham group, and 310 proteins significantly changed (157 increased, 153 decreased) in response to salt load in 5/6Nx animals. In our global phosphoproteomic study, 1810 unique phosphopeptides including 2565 non-redundant phosphosites were identified in renal cortex of 5/6Nx rats, which corresponded to 550 phosphoproteins. By comparing normal salt-fed 5/6Nx group with sham group, 113 phosphopeptides upregulated and 84 phosphopeptides downregulated. High salt intake upregulated 78 phosphopeptides and downregulated 91 phosphopeptides relative to normal salt intake in 5/6Nx rats. The differentially phosphorylated proteins were classified into catalytic molecules, binding proteins transporters, structural molecules, and signaling molecules, which were involved in metabolic process, transport, response to stimulus, cell proliferation and others. Although the pathological link of these phosohopeptides and phosphoproteins with renal failure needs to be further verified, profiling renal phosphoproteome associated with advanced kidney damage and salt intake will have significant implications for the pathogenesis and therapeutic targets of chronic kidney disease and salt sensitivity.

## Materials and Methods

### Ethics Statement

All studies using animals were reviewed and approved by the Institutional Animal Care and Use Committee of Nanfang Hospital at Southern Medical University and in accordance with the ARRIVE guidelines^65^. The study protocol was reviewed and approved by the Ethics Committee for experimental animals, Southern Medical University (No. 0064713, 1838 North Guangzhou Avenue, Guangzhou, China).

### Animals

Male Sprague-Dawley rats weighing 180 to 200 g were obtained from Animal Experiment Center at Nanfang Hospital, and maintained under *ad libitum* feeding conditions on a 12 h light-12 h dark cycle at room temperature.

Subtotal nephrectomy was carried out under sodium pentobarbital (35 mg/kg, i.p.) anesthesia and in sterile conditions. Rats with five-sixths nephrectomy are a widely studied model of advanced kidney damage^66^. The rats underwent 2/3 nephrectomy of the left kidney and, 1 week later, total nephrectomy of the right kidney, for a final reduction of 5/6 of the total renal mass. Age-matched rats undergone sham operations were used as controls. Blood pressure measurements were performed before surgery and every two weeks thereafter by the tail cuff method. Blood samples were drawn from the tail vein of the animals before and 10 week after 5/6 nephrectomy for measurement of biochemical parameters. Urinary samples were collected 10 week after 5/6 nephrectomy for measurement of urinary protein excretion.

### Salt diet administration

Ten weeks after surgery, 5/6 nephrectomy model of advanced kidney damage and sham rats were randomly assigned to 3 groups and received the following treatments, respectively: (1) sham rats fed normal-salt diet (0.4% NaCl, wt/wt) (Sham + NS, n = 6); (2) 5/6Nx rats fed normal-salt diet (0.4% NaCl, wt/wt) (5/6Nx + NS, n = 6); (3) 5/6Nx rats fed high-salt diet (4% NaCl, wt/wt) (5/6Nx + HS, n = 6). The rats were fed commercially available rat chow containing different concentrations of salt (TROPHIC Animal Feed High-Tech Co., Ltd, Nantong, China) for 2 weeks. After salt diet administration, blood pressure measurements were performed and blood and urinary samples were collected^18^.

### Renal Function and Blood Pressure Measurement

Serum creatinine and 24-h urine sodium excretion was measured by a Hitachi 7600 autoanalyzer (Hitachi, Japan). The 24-h urinary protein excretion was determined with the Coomassie Blue method^67^. Blood pressure was quantified by using a tail cuff sphygmomanometer (BP-98A, softron, Japan) before and after salt treatment. Systolic blood pressure measurement was performed 5 or 6 times and the values were averaged, as described previously^18^.

### Protein Extraction

Proteins for proteomic and phosphoproteomic analysis were extracted and processed as described in detail previously^18^. Approximately 2 g frozen renal cortex tissues from an equal amount of 6 different rats in each group were dissected, ground, homogenized in lysis buffer [4% SDS, 1 mM DTT, 150 mM Tris-HCl, pH 8], incubated for 3 min in boiling water, and sonicated on ice. The homogenates were incubated again and centrifugated. Concentrations of the protein supernatants were determined with the Bicinchoninic acid protein assay reagent (Beyotime, China).

### Protein Digestion and iTRAQ Labeling

Protein was digested based on the FASP procedure established by Wisniewski *et al.*^68^ and digestion mixture was labeled with the 8-plex iTRAQ reagent (Applied Biosystems) following the instructions of the manufacturer as described previously^18^. Briefly, 200 μg of proteins for each group were resuspended in 30 μl of standard buffer (4% SDS, 100 mM DTT, 150 mM Tris-HCl pH 8.0). The detergent, DTT and other low-molecular-weight components were removed using uric acid buffer (8 M Urea, 150 mM Tris-HCl, pH 8.0) by repeated ultrafiltration (Microcon units, 30 kD). Then 100 μl 0.05 M iodoacetamide in uric acid buffer was used to block reduced cysteine residues. After incubation for 20 min in darkness, the samples were washed with 100 μl uric acid buffer three times and then 100 μl DS buffer (50 mM triethylammonium bicarbonate at pH 8.5) twice. The protein suspensions were finally digested with 2 μg trypsin (Promega) in 40 μl DS buffer overnight at 37 °C, and the resulting peptides were collected as a filtrate. The peptide content was estimated by ultravlolet light at 280 nm using an extinction coefficient of 1.1 of 0.1% (g/l) solution that was calculated according to the frequency of tryptophan and tyrosine in vertebrate proteins.

For labeling, each iTRAQ reagent was dissolved in 70 μl of ethanol and added to the respective peptide mixture. The samples from normal salt-fed sham (Sham + NS), normal salt-fed 5/6Nx (5/6Nx + NS) and high salt-fed 5/6Nx groups (5/6Nx + HS) were labeled with iTRAQ tags 113, 114 and 115, respectively, multiplexed and vacuum dried.

### Enrichment of phosphorylated peptiedes

Digested peptides were enriched for phosphopeptides using titanium dioxide (TiO_2_) as described in detail previously^18^. The concentrated final peptide mixture was resuspended in loading buffer (2% glutamic acid/65% acetonitrile/2% trifluoroacetic acid). After TiO_2_ beads were added, the peptide mixture were agitated and centrifuged. TiO_2_ beads washed with washing buffer I (30% acetonitrile/3% trifluoroacetic acid) and then washing buffer II (80% acetonitrile/0.3% trifluoroacetic acid) to remove the remaining non-adsorbed materials. The phosphopeptides were eluted with elution buffer (40% acetonitrile/15% ammonium hydroxide) and lyophilizated.

### Mass spectrometry

Experiments were performed on a Q Exactive mass spectrometer that was coupled to Easy nano-liquid chromatography (nLC) (Proxeon Biosystems, now Thermo Fisher Scientific). Ten microliters of each fraction was injected for nano-liquid chromatography–tandem mass spectrometry (nano-LC-MS/MS) analysis as described previously^18^. Five microliters of the peptides before TiO_2_ enrichment or phosphopeptides solution mixed with 15 ul 0.1% (v/v) trifluoroacetic acid was subjected to nano-liquid chromatography–tandem mass spectrometry (nano-LC-MS/MS) analysis as described previously^18^. Liquid chromatography solvents were 0.1% formic acid in H_2_O (buffer A) and 0.1% formic acid in acetonitrile (buffer B). The peptide solutions were injected into a C18-reversed phase column in buffer A and separated with a linear gradient of buffer B. Flow rate was 250 nl/min. After injection, a 240 gradient from 0% buffer B to 100% buffer B was used to elute the peptides from the column.

MS data was acquired using a data-dependent top10 method^18^. A full mass spectrometry scan (300–1800m/z) for higher energy collisional (C-trap) dissociation fragmentation was acquired in the positive ion mode. Determination of the target value is based on predictive Automatic Gain Control (pAGC), which is 3–6. Dynamic exclusion duration was 40.0s. Survey scans were acquired at a resolution of 70,000 at m/z 200. Resolution for higher energy collisional (C-trap) dissociation spectra was set to 17,500 at m/z 200. Normalized collision energy was set to 27 eV. The under fill ratio, which specifies the minimum percentage of the target value likely to be reached at maximum fill time, was defined as 0.1%. The instrument was run with peptide recognition mode enabled.

### Data processing

MS/MS spectra were analyzed using the software Mascot version 2.2 (Matrix Science, London, UK) against the Uniprot database and the reversed database. The following search parameters were set for database search: Peptide mass tolerance, ±20 ppm; fragment mass tolerance, 0.1 Da; enzyme, trypsin; mass values, monoisotopic; max missed cleavage, 2; fixed modification, carbamidomethyl (C), variable modification: oxidation (M); phosphorylation (S/T/Y). Peptide false discovery rate (FDR) ≤0.01 and a peptide Mascot score >20 were defined as significant.

The phosphorylation peptides were analyzed using Proteome Discoverer 1.3 (Thermo Electron, San Jose, CA.). pRS score above 50 indicate a good PSM (Peptide Spectrum Matches) and pRS probabilities above 75 percent indicate that a site is unambiguously localized with high confidence^69^.

### Statistical analysis

All data are expressed as means ± SEM of these independent experiments. Continuous variables between groups were compared using one-way ANOVA, followed by LSD test when p < 0.05. All analyses were performed using SPSS 13.0 for Windows (SPSS, Chicago, IL). A value of p < 0.05 was defined statistical significant for all analyses.

### Western blot analysis

Western blot analyses were carried out as previously described^56^. The primary antibodies anti-Phospho-phospholamban (Ser16, Cell Signaling, Beverly, MA, USA), anti-phospholamban (Abcam, Cambridge, MA, USA), anti-Phospho-lamin A (Ser22, Santa Cruz Biotechnology, Santa Cruz, CA, USA), anti-lamin A (Abcam) and anti-β-actin (Cell Signaling) were used.

### Real-time reverse transcriptase-polymerase chain reaction

Total RNA was extracted from rat renal cortexes using Trizol reagent (Invitrogen) according to the manufacturer’s instruction. The first strand of complementary DNA was synthesized using 1 mg of RNA in 20 ml of reaction buffer using M-MLV reverse transcriptase (Invitrogen). Real-time PCR was performed using the SYBR^®^Premix Ex Taq™ kit (TaKaRa, Kyoto, Japan) and the Fast Real-Time PCR system 7500 (Applied Biosystems, CA). The primer sequences were as follows: desmin forward: 5′-GGG CGA GGA GAG CCG GAT CA-3′, reverse: 5′-TCC CCG TCC CGG GTC TCA ATG-3′; SERCA2 forward: 5′-AAG CAG TTC ATC CGC TAC CT-3′, reverse: 5′-AGA CCA TCC GTC ACC AGA TT-3′; GADPH forward: 5′-GGG TGT GAA CCA CGA GAA AT-3′, reverse: 5-ACT GTG GTC ATG AGC CCT TC-3′. GAPDH RNA was amplified to normalize differences in RNA amounts.

## Additional Information

**How to cite this article**: Jiang, S. *et al.* Proteomic and phosphoproteomic analysis of renal cortex in a salt-load rat model of advanced kidney damage. *Sci. Rep.* **6**, 35906; doi: 10.1038/srep35906 (2016).

## Supplementary Material

Supplementary Information

Supplementary Dataset 1

Supplementary Dataset 2

Supplementary Dataset 3

Supplementary Dataset 4

Supplementary Dataset 5

Supplementary Dataset 6

## Figures and Tables

**Table 1:** Physiological and metabolic parameters in sham and 5/6Nx rats at week 12 after surgery^A^.

	Sham + Normal salt	5/6Nx + Normal salt	5/6Nx + High salt
Body weight (g)	504.2 ± 11.7	470 ± 12.3	475 ± 11.1
KW/BW (mg/g)	3.1 ± 0.2	4.3 ± 0.2^B^	4.7 ± 0.1^B,C^
SBP (mmHg)	125.6 ± 3.1	140.6 ± 1.9^B^	154.8 ± 2.8^B,C^
SCr (μmol/l)	73.1 ± 6.1	111.4 ± 7.8^B^	113.6 ± 10.8^B^
Urine Na + (μmol/24h)	856.3 ± 59.4	763.7 ± 53.8	10708 ± 699.2^B,C^
UPE (mg/24h)	10.44 ± 0.64	15.34 ± 0.81^B^	42.39 ± 2.48^B,C^

^A^Data from 3 independent experiments are expressed as mean ± SD (n = 6 in each group).

^B^*p* < 0.05 *vs.* rats fed with normal salt in the sham group.

^C^*p* < 0.05 *vs.* 5/6Nx group fed with normal salt diet. KW/BW, kidney weight/body weight. SBP, systolic blood pressure; SCr, serum creatinine; UPE, urinary protein excretion.

**Table 2:** List of proteins significantly differentially expressed at least1.5-fold corresponding to peptides identified by LC-MS/MS analysis of renal cortex samples from both 5/6Nx and sham rats fed normal or high salt diet.

Accession	Description	MW [kDa]	Molecular Function	Comparison between groups			
				(5/6Nx + NS)/(Sham + NS)		(5/6Nx + HS)/(5/6Nx + NS)	
				Ratio	Log10*p*-value	Ratio	Log10*p*-value
IPI00951759.1	procollagen, type VII, alpha 1	259	protein binding	1.78	−8.93E + 00	0.27	−9.37E + 01
IPI00190035.1	Tuftelin-interacting protein 11	96	DNA binding	1.64	−6.69E + 00	0.34	−6.29E + 01
IPI00191866.6	ADP-ribosylation factor-like protein 6	21	nucleotide binding; signal transducer activity	0.37	−3.45E + 01	1.95	−3.26E + 01
IPI00231350.5	Parvalbumin alpha	12	metal ion binding	1.59	−6.05E + 00	0.52	−2.43E + 01
IPI00421302.4	Uncharacterized protein	58	catalytic activity; protein binding	0.46	−2.12E + 01	1.91	−3.06E + 01
IPI00369236.3	Transducin beta-like protein 3	88	protein binding	1.76	−8.56E + 00	0.61	−3.60E + 00
IPI00560241.3	LRRGT00098	143	nucleotide binding	0.48	−3.60E + 00	2.25	−4.75E + 01
IPI00231191.7	Glutaredoxin-1	12	catalytic activity	1.71	−7.87E + 00	0.70	−7.55E + 00
IPI00569001.1	estradiol 17-beta-dehydrogenase 12	32	catalytic activity; nucleotide binding	1.78	−8.89E + 00	0.71	−7.19E + 00
IPI00231099.3	Secreted phosphoprotein 24	23	enzyme regulator activity	1.56	−5.54E + 00	0.83	−2.34E + 00
IPI00957367.1	urocanase domain containing 1-like	71	catalytic activity	0.83	−1.64E + 00	1.62	−1.75E + 01
IPI00951003.1	Uncharacterized protein	71	metal ion binding	2.15	−1.51E + 01	0.63	−1.25E + 01
IPI00767285.3	protocadherin beta 18	87	metal ion binding	1.56	−5.51E + 00	0.93	−6.47E-01
IPI00198021.3	Uromodulin	71	metal ion binding; protein binding	1.56	−5.52E + 00	0.98	−1.13E-01
IPI00211916.1	Integral membrane protein 2B	30	nucleotide binding; protein binding	1.58	−5.81E + 00	0.83	−2.40E + 00
IPI00778544.2	DEAD (Asp-Glu-Ala-Asp) box polypeptide 58	68	catalytic activity; DNA binding; metal ion binding; nucleotide binding	0.93	−4.43E-01	1.60	−1.68E + 01
IPI00363506.1	Uncharacterized protein	29	DNA binding	1.56	2.96E-06	0.97	−2.10E-01
IPI00211999.2	mRNA cap guanine-N7 methyltransferase	46	catalytic activity; RNA binding	0.93	−4.11E-01	1.63	−1.77E + 01
IPI00959049.1	rCG32673-like	90		2.43	−2.01E + 01	0.63	−1.27E + 01
IPI00393763.2	Vomeronasal V1r-type receptor V1rc2	36	receptor activity; signal transducer activity	0.97	−1.58E-01	1.59	−1.61E + 01
IPI00950436.2	Uncharacterized protein	65	protein binding	1.50	−4.77E + 00	1.06	−5.78E-01
IPI00371221.5	small G protein signaling modulator 1	123	enzyme regulator activity	1.07	−3.29E-01	1.62	−1.76E + 01
IPI00368797.2	Cardiolipin synthase	33	catalytic activity	1.69	−7.46E + 00	0.92	−7.16E-01
IPI00559310.2	Uncharacterized protein (Fragment)	59	receptor activity; signal transducer activity	1.68	−7.31E + 00	0.97	−1.96E-01
IPI00206975.3	Nitric oxide synthase, endothelial	133	catalytic activity; metal ion binding; nucleotide binding; protein binding	0.75	−3.60E + 00	2.19	−4.44E + 01
IPI00206995.2	Cytochrome P-450c	60	catalytic activity; metal ion binding	0.98	−8.58E-02	1.73	−2.23E + 01
IPI00191737.6	Serum albumin	69	DNA binding; metal ion binding; protein binding	1.55	−5.45E + 00	1.14	−1.78E + 00
IPI00211927.1	Lysozyme C-1	17	catalytic activity	2.09	−1.42E + 01	0.88	−1.31E + 00
IPI00231852.1	Isoform Stat5A2 of Signal transducer and activator of transcription 5A	85	metal ion binding; protein binding; signal transducer activity	1.05	−2.38E-01	2.37	−5.37E + 01
IPI00768731.2	signal recognition particle 14-like	18	RNA binding	1.13	−6.93E-01	2.25	−4.74E + 01
IPI00780911.2	Uncharacterized protein	50	protein binding	2.46	−2.07E + 01	1.04	−3.38E-01
IPI00370496.4	Isoform 1 of Protein QN1 homolog	161		2.51	−2.14E + 01	1.03	−2.14E + 01
IPI00779176.2	Uncharacterized protein	55	transporter activity	1.15	−8.37E-01	2.39	−5.49E + 01
IPI00766706.1	serine/threonine-protein kinase MARK2-like	73	catalytic activity; nucleotide binding	2.64	−2.38E + 01	1.04	−3.03E-01

n = 3 independent replicates.

MW, Molecular weight. Sham + NS, sham operation + normal salt; 5/6Nx + NS, 5/6Nx + normal salt; 5/6Nx + HS, 5/6Nx + high salt.
