# Supplementary material for: Proteomic and phosphoproteomic analysis of renal cortex in a salt-load rat model of advanced kidney damage
Source: Sci Rep. 2016 Oct 24;6:35906. doi: 10.1038/srep35906 (PMC5075906; doi:10.1038/srep35906)
Supplement: Supplementary Information [file srep35906-s1.pdf]

Supplementary file 1 for
Proteomic and phosphoproteomic analysis of renal cortex in a salt-load rat model of advanced kidney damage

Shaoling Jiang[1#], Hanchang He[2#], Lishan Tan[1], Liangliang Wang[3], Zhengxiu Su[1], Yufeng Liu[1], Hongguo Zhu[1], Menghuan Zhang[1], Fan Fan Hou[1], Aiqing Li[1*]

[1]State Key Laboratory of Organ Failure Research, National Clinical Research Center of Kidney Disease, Division of Nephrology, Nanfang Hospital, Southern Medical University, Guangzhou, China; [2]The First People's Foshan Hospital, Foshan, China; and [3]Division of Nephrology, First Affiliated Hospital of Guangzhou University of Traditional Chinese Medicine, Guangzhou 510405, P.R. China

[#]Shaoling Jiang and Hanchang He contributed equally to this work.

*Correspondence: Aiqing Li, Phone: 86-20-62787973; FAX: 86-20-87281713; E-mail: liaiqing@smu.edu.cn

This file includes:

Figures S1-S4; Table S1

Other Supporting information for this manuscript (not in this file) includes:

Supplementary Datasets 1-6

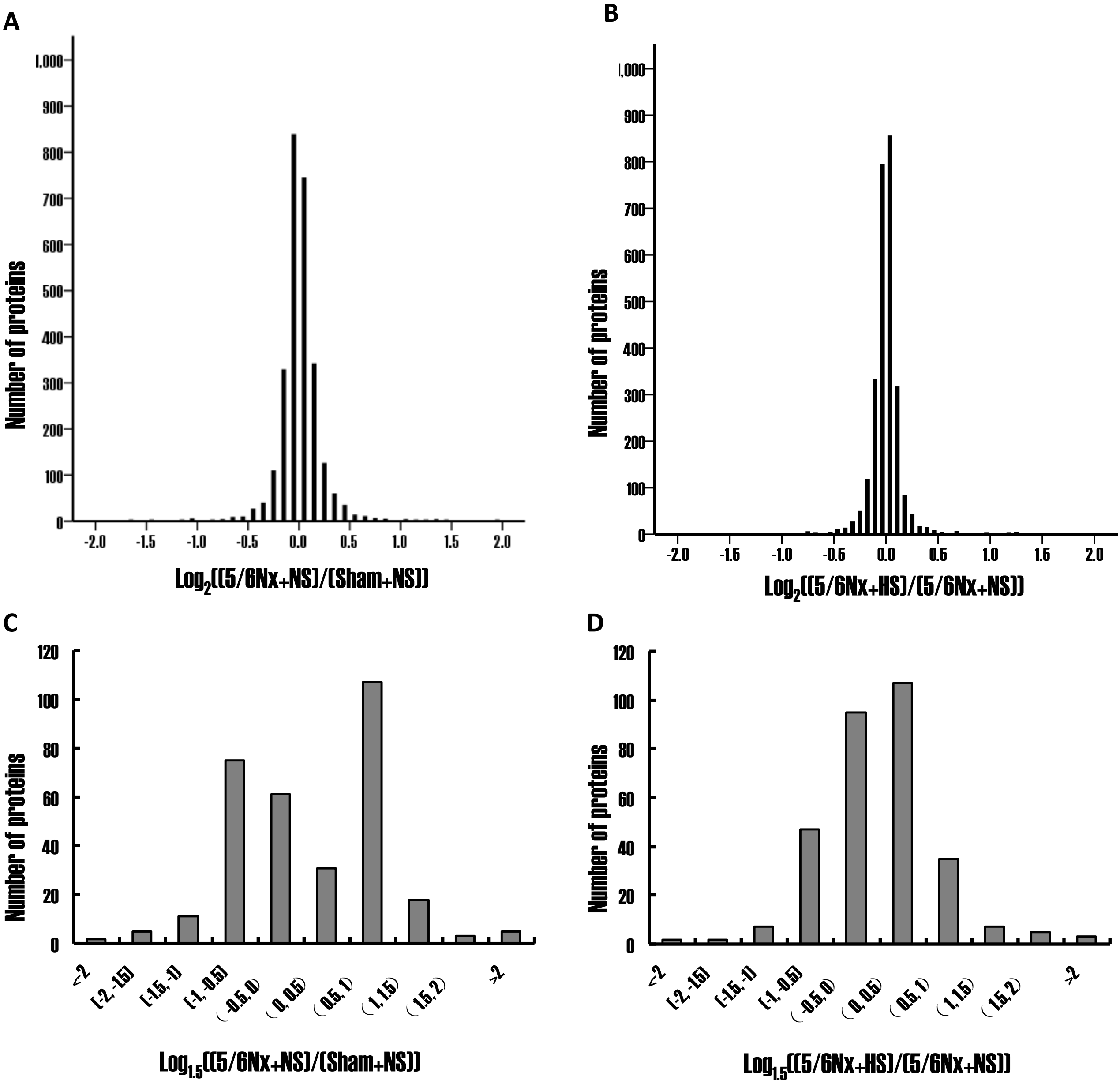

Figure S1. Quantification of identified renal proteins before phosphopeptide enrichment in sham or 5/6Nx rats. (A & B) The distribution of the $\log_2$ (5/6Nx+NS)/(Sham+NS) (A) or (5/6Nx+HS)/(5/6Nx+NS) (B) ratio for abundance of all identified proteins. (C & D) Frequency distribution of Relative quantification of significantly altered proteins expressed as a $\log_{1.5}$ (5/6Nx+NS)/(Sham+NS) (C) or (5/6Nx+HS)/(5/6Nx+NS) (D) ratio. Sham operation+normal salt; 5/6Nx+NS, 5/6Nx+normal salt; Sham+NS, 5/6Nx+HS, 5/6Nx+high salt.

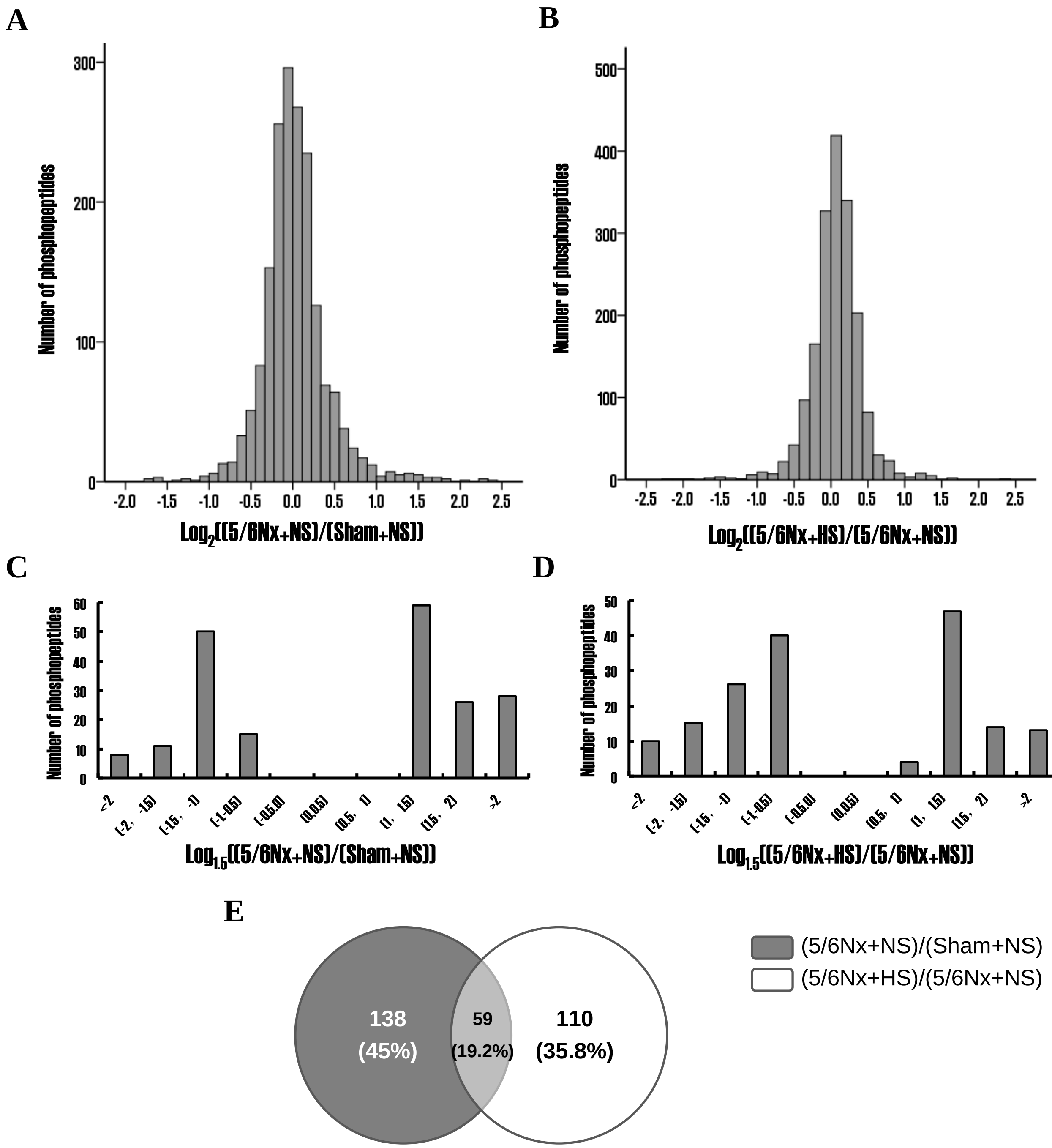

Figure S2 Characterization of differentially phosphorylated peptides and proteins. (A, B) Frequency distribution of relative abundance of identified phosphopeptide at the Log2 (5/6Nx+NS)/(Sham+NS) or Log2 (5/6Nx+HS)/(5/6Nx+NS) ratio; (C, D) Frequency distribution of differentially phosphorylatedpeptides at the log1.5 (5/6Nx+NS)/(Sham+NS) or (5/6Nx+HS)/(5/6Nx+NS) ratio. (E) Venn diagram analyses of the coordinatially regulated phosphopeptides by chronic kidney disease and high salt. Sham+NS, Sham operation+normal salt; 5/6Nx+NS, 5/6Nx+normal salt; 5/6Nx+HS, 5/6Nx+high salt.

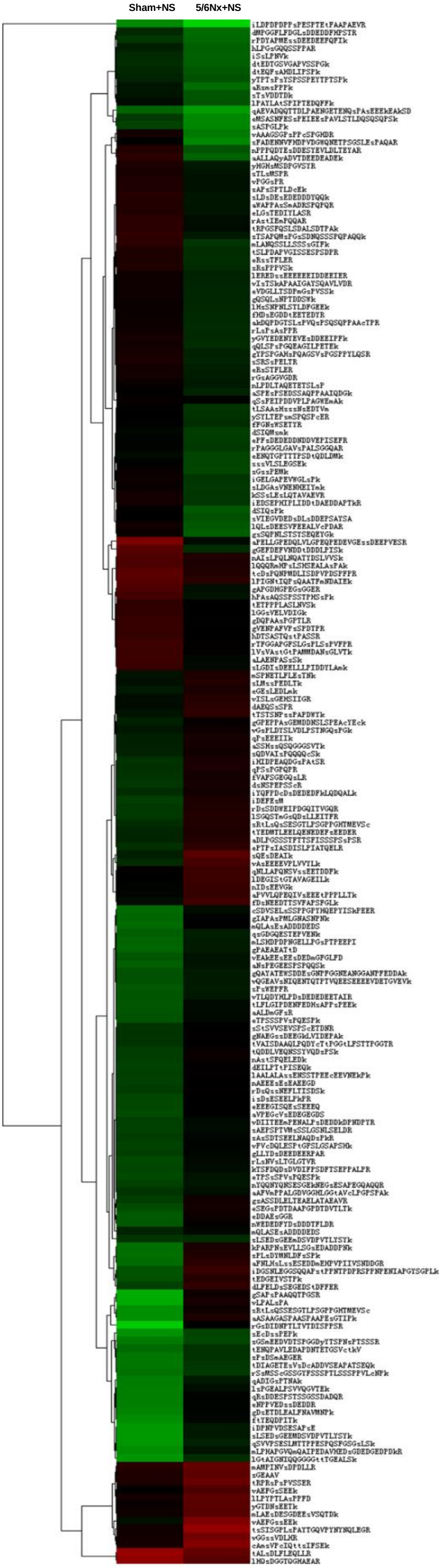

Figure S3. Cluster analysis of the differentially phosphorylated peptides between 5/6Nx+NS **and** Sham+NS **groups using Cluster software.** The 197 phosphopeptides are clustered on the basis of the relative abundance ratio. Increased and decreased abundance are colored in red and green, respectively. Sham+NS, Sham operation+normal salt; 5/6Nx+NS, 5/6Nx+normal salt;

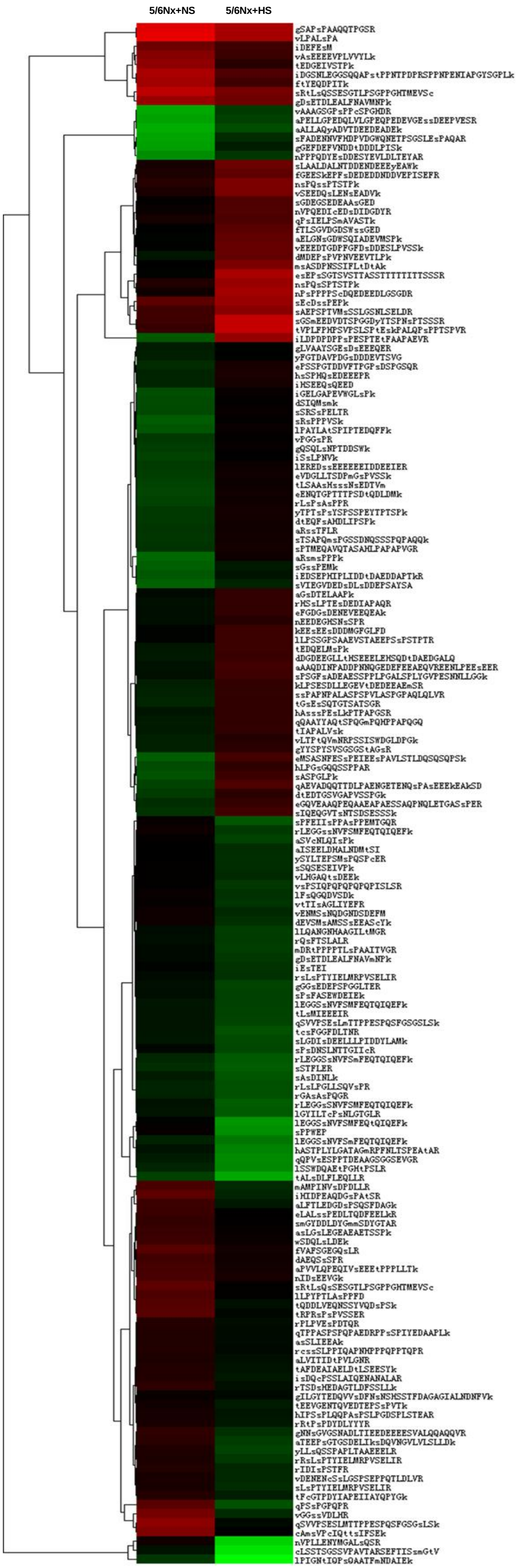

Figure S4 Cluster analysis of the differentially phosphorylated peptides between 5/6Nx+NS **and** 5/6Nx+HS **groups using Cluster software.** The 169 phosphopeptides are clustered on the basis of the relative abundance ratio. Increased and decreased abundance are colored in red and green, respectively. 5/6Nx+NS, 5/6Nx+normal salt; 5/6Nx+HS, 5/6Nx+high salt.

Table S1. Physiological and metabolic parameters in rats at week 10 after sham or 5/6 nephrectomy operation[A]

	Sham+Normal salt	5/6Nx+ Normal salt	5/6Nx + High salt
Body weight (g)	467.7±3.8	451.0±4.4	456.0±8.0
SBP (mmHg)	124.9±2.6	136.8±3.8 [B]	137.0±3.1 [B]
SCr (μmol/l)	71.7±2.7	107.0±10.5 [B]	110.8±3.0 [B]
BUN (mmol/l)	3.5±0.9	9.3±0.7 [B]	9.1±0.9 [B]
UPE (mg/24h)	12.3±0.8	22.2±1.3 [B]	21.9±1.4 [B]

[A] Data from 3 independent experiments are expressed as mean ± SD (n = 6 in each group); [B] $p < 0.05$ *vs.* (sham+Normal salt) group; SBP, systolic blood pressure; SCr, serum creatinine; BUN, blood urea nitrogen; UPE, urinary protein excretion.
